# CMR‐derived skeletal muscle T1 time and extracellular volume as novel diagnostic markers for cardiac amyloidosis

**DOI:** 10.1111/eci.70191

**Published:** 2026-03-30

**Authors:** Christina Kronberger, Katharina Mascherbauer, Michael Poledniczek, Lena Marie Schmid, Carolina Donà, Matthias Koschutnik, Laura Lunzer, René Rettl, Franz Duca, Christina Binder, Christian Nitsche, Nikita Ermolaev, Gözde Celebi, Varius Dannenberg, Dietrich Beitzke, Roza Badr Eslam, Jutta Bergler‐Klein, Julia Mascherbauer, Johannes Kastner, Christian Hengstenberg, Andreas Anselm Kammerlander

**Affiliations:** ^1^ Division of Cardiology, Department of Internal Medicine II Medical University of Vienna Vienna Austria; ^2^ Division of Cardiovascular and Interventional Radiology, Department of Biomedical Imaging and Image‐Guided Therapy Medical University of Vienna Vienna Austria; ^3^ Karl Landsteiner University of Health Sciences, Department of Internal Medicine 3 University Hospital St. Pölten Krems Austria

**Keywords:** cardiac amyloidosis, diagnosis, ECV, native T1 time, T1‐mapping

## Abstract

**Introduction:**

Cardiac amyloidosis (CA) is a progressive cardiomyopathy caused by amyloid deposition, leading to heart failure and increased mortality. Cardiac magnetic resonance (CMR) identifies myocardial involvement via elevated native T1 relaxation time and extracellular volume (ECV). Although amyloid infiltration has also been observed in thoracic skeletal muscle, the diagnostic and prognostic relevance of thoracic skeletal muscle T1 time and ECV remain unclear.

**Aim:**

To compare native thoracic skeletal muscle T1 time and ECV between CA patients and controls and assess their diagnostic and prognostic value.

**Methods:**

In a prospective CMR registry, consecutive CA patients and controls underwent CMR with T1‐mapping. Native and post‐contrast T1 relaxation time and ECV were quantified in myocardium and thoracic skeletal muscles. Diagnostic performance was evaluated using ROC analysis and associations with mortality were assessed using Cox regression.

**Results:**

Among 1976 participants (267 CA, 1709 controls), CA patients showed significantly higher native myocardial T1 time and ECV, as well as elevated native thoracic skeletal muscle T1 time (919.4 vs. 868.5 ms, *p* < 0.001) and ECV (16.4 vs. 12.9%, p < 0.001). Native thoracic skeletal muscle T1 time demonstrated moderate diagnostic performance for CA (AUC = 0.70), with an optimal cutoff of 895 ms yielding a sensitivity of 61% and specificity of 59%. Higher native thoracic skeletal muscle T1 time predicted increased mortality (HR = 1.65 per 100 ms, *p* < 0.001), and this association remained significant after adjusting for age, sex and ventricular function (HR = 1.21 per 100 ms, *p* = 0.008).

**Conclusion:**

Native skeletal muscle T1 relaxation time and ECV indicate systemic amyloid involvement and provide additional diagnostic and prognostic information beyond myocardial assessment, potentially supporting improved detection and risk stratification in CA.

## Graphical abstract

This study compared T1 time and ECV in cardiac and thoracic skeletal muscle using CMR in 267 CA patients and 1709 non‐CA controls. Representative T1‐maps (central panel) illustrate regions of interest placed in the cardiac and thoracic skeletal muscle. Arrow markers on the adjacent scales (right side of the figure) indicate the median native T1 times measured in cardiac and thoracic skeletal muscle tissue. CA patients exhibited significantly higher native T1 times and ECV in both cardiac and thoracic skeletal muscle, suggesting amyloid infiltration extends beyond the heart to involve skeletal muscles. Native thoracic skeletal muscle T1 time and ECV may serve as novel noninvasive markers for detecting CA. CA indicates cardiac amyloidosis; CMR, cardiac magnetic resonance; ECV, extracellular volume.

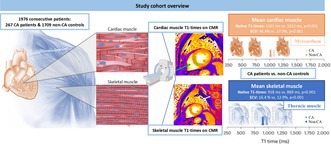

## 1 INTRODUCTION

Cardiac amyloidosis (CA) is a progressive disease characterized by the extracellular deposition of amyloid fibrils in the myocardium,^1^ resulting in progressive heart failure, arrhythmias and ultimately death.^2^ The two main subtypes of CA are light chain (AL) and transthyretin (ATTR) amyloidosis. ATTR‐CA can be further classified into hereditary (variant ATTR‐CA), caused by mutations in the transthyretin gene, and wild‐type ATTR‐CA, an acquired form primarily affecting older adults.^2^, ^3^

Despite recent advancements in understanding CA pathogenesis and the emergence of specific pharmacological therapies, amyloidosis remains difficult to diagnose due to its broad spectrum of clinical manifestations and nonspecific symptoms, which vary depending on the organs involved. This diagnostic complexity often leads to delays in diagnosis and treatment initiation, thereby adversely affecting patient outcomes. Early and accurate diagnosis is crucial for initiating targeted therapies that can slow disease progression and improve patient outcomes.^4^

Cardiac magnetic resonance (CMR) imaging has emerged as a key noninvasive diagnostic tool in CA. Elevated native myocardial T1 time and increased ECV are highly characteristic of CA, with values higher than those found in any other cardiomyopathy.^5^ These markers strongly correlate with disease severity, providing both diagnostic and prognostic value.^6^ However, in early disease stages, myocardial involvement can be subtle or indistinct, making differentiation from other myocardial pathologies challenging. Since CA is a systemic disease,^7^ amyloid deposition may also affect skeletal muscles, an often‐overlooked yet common manifestation^8^ that could serve as an early indicator of systemic amyloid burden.^9^ Importantly, skeletal muscles are already visualized on standard cardiac T1‐maps, enabling the simultaneous evaluation of both myocardial and thoracic skeletal muscle tissue changes without the need for additional imaging protocols.

Magnetic resonance‐based skeletal muscle imaging has been used in neuromuscular diseases, where elevated native T1 time in lower limb muscles has been shown to reflect pathological changes.^10^, ^11^ Similarly, studies have identified skeletal muscle abnormalities, such as hyperintensity and hypertrophy of lower limb muscles, in patients with systemic amyloidosis,^12^, ^13^, ^14^ indicating that amyloid deposition in skeletal muscle may be detectable by CMR. However, the diagnostic utility of native thoracic skeletal muscle T1 time and ECV in CA remains unexplored.

This study aims to investigate native thoracic skeletal muscle T1 time and ECV in patients with CA compared to non‐CA controls and assess their diagnostic and prognostic utility. We hypothesize that compared to controls, patients with CA exhibit elevated native thoracic skeletal muscle T1 time and ECV, reflecting amyloid deposition in skeletal muscle.

## 2 METHODS

### 2.1 Study design and participants

This cohort study was conducted within the framework of a prospective CMR registry at the Medical University of Vienna, a university‐affiliated tertiary care center and national referral center for CA. The center features a dedicated CA clinic and advanced CMR imaging facilities. Between July 2012 and May 2025, consecutive all‐comer referrals undergoing CMR were systematically screened for inclusion and categorized into two groups: (1) CA patients, subdivided into AL and ATTR (hereditary and wild‐type) subtypes, and (2) a control group, consisting of patients undergoing CMR for various cardiac conditions but without clinical or imaging evidence of CA.

Patients aged 18 years or older were eligible for inclusion. Patients with known severe myopathies or severe peripheral edema at the time of CMR were excluded.

Demographic information, clinical history, laboratory findings and CMR imaging data were prospectively collected for all participants. Mortality was recorded during follow‐up. The study was approved by the local ethics committee (EK#2036/2015) and conducted in accordance with the principles outlined in the Declaration of Helsinki. Written informed consent was obtained from all participants prior to their inclusion in the study.

### 2.2 Diagnosis of CA

CA was diagnosed in accordance with the latest European Society of Cardiology guidelines for cardiomyopathies.^15^

ATTR‐CA was confirmed noninvasively by a Perugini grade ≥2 myocardial tracer uptake on [^99m^Tc]‐radiolabeled diphosphono‐1,2‐propanodicarboxylic acid scintigraphy, with exclusion of a monoclonal protein via serum and urine immunofixation,^16^ or invasively by Congo red–positive endomyocardial biopsy showing apple‐green birefringence and TTR immunoreactivity. Genetic testing was performed to differentiate patients with hereditary from those with wild‐type ATTR‐CA.

AL‐CA was diagnosed by detection of a monoclonal protein in serum or urine together with typical cardiac imaging findings and histological confirmation of AL amyloid deposits in either a noncardiac or endomyocardial biopsy.

### 2.3 CMR imaging protocol

CMR scans were performed using a 1.5‐T whole‐body scanner (Avanto Fit, Siemens Healthcare GmbH, Erlangen, Germany) following standardized protocols.^17^, ^18^ The imaging protocol included late gadolinium enhancement imaging with gadobutrol (Gadovist, Bayer Vital GmbH, Leverkusen, Germany) and T1‐mapping before and after contrast administration to determine the myocardial and skeletal muscle T1 relaxation time.

T1‐mapping was conducted using the Modified Look‐Locker Inversion recovery sequence in short‐axis and four‐chamber views. Native T1‐maps were acquired using a 5b(3b)3b scheme (five acquisition heartbeats are followed by three recovery heartbeats and then three acquisition heartbeats; b stands for heartbeat). Post‐contrast T1‐maps were obtained 15 min after gadolinium administration using a 4b(1b)3b(1b)2b scheme. The comprehensive CMR protocol also included standard short‐axis and long‐axis cine imaging for assessing cardiac morphology and function.

### 2.4 CMR image analysis

All images were analysed by an experienced investigator blinded to the patient's data using offline post‐processing software (IMPAX EE R20 XV, Agfa Healthcare). To assess inter‐observer variability, a second investigator independently measured native thoracic T1 time in a random subset of 165 patients.

**FIGURE 1:**
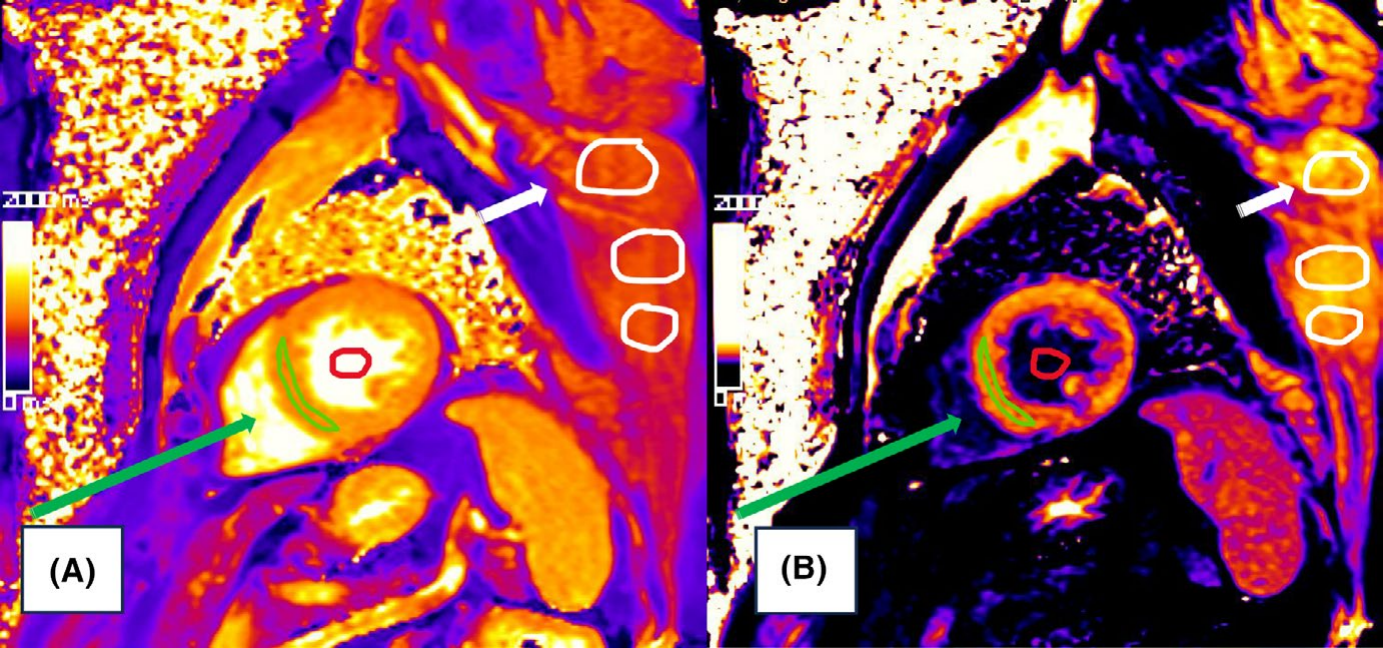
Representative T1‐maps displaying cardiac and skeletal muscle. (A, left) Native and corresponding (B, right) post‐contrast T1‐map illustrating manual contour tracing for T1 time measurement (in milliseconds). Regions of interest include the left ventricular myocardium (green), the blood pool (red) and skeletal muscle (white circles), enabling simultaneous assessment of myocardial and skeletal muscle tissue characteristics.

Native and post‐contrast thoracic T1 times were determined by manually drawing regions of interest (ROI) in the left ventricular myocardium, blood pool and thoracic skeletal muscle on mid‐ventricular short‐axis slices before and after contrast administration (Figure 1).

For thoracic skeletal muscle, three ROIs were placed within visually homogeneous tissue in the upper, middle and lower portions of the rotator cuff musculature (infraspinatus or subscapularis, depending on individual anatomy), and the average native and post‐contrast T1 time was calculated. As the field of view was optimized for cardiac assessment, only one side of the thoracic musculature was consistently visualized and measured. Partial‐volume effects were minimized by avoiding muscle borders and by excluding areas adjacent to fat, bone, lung tissue, focal fat infiltration, signal inhomogeneity or artefacts. Myocardial ROIs were drawn, avoiding endocardial and epicardial borders, and blood pool T1 was measured centrally in the left ventricle at a sufficient distance from papillary muscles and endomyocardial borders.

ECV for both myocardium and thoracic skeletal muscle was calculated using pre‐native and post‐contrast T1 time and haematocrit values according to the established formula^19^:

$$\mathrm{ECV}=\left(1-\text{hematocrit}\right)\times \frac{\Delta R1_{\text{tissue}}}{\Delta R1_{\text{blood}}},\Delta R1=\frac{1}{T1_{\text{post}}}-\frac{1}{T1_{\text{native}}}$$

ΔR1 represents the change in relaxation rate after contrast administration. The formula was applied separately for each tissue, myocardium (using myocardial T1 time) and thoracic skeletal muscle (using skeletal muscle T1 time), with the same blood pool T1 from the left ventricular cavity serving as reference for both calculations. Haematocrit values were obtained from routine blood tests.

### 2.5 Propensity score matching

To address baseline confounding, we performed 1:1 nearest‐neighbour propensity score matching on age, sex, estimated glomerular filtration rate (eGFR), log‐transformed N‐terminal prohormone of brain natriuretic peptide (NT‐proBNP) and atrial fibrillation status. Following recommendations for high‐quality matching,^20^ we tested calliper widths of 0.1 and 0.2 standard deviations (SD) of the logit propensity score. Both callipers produced identical matches (234 CA patients, 114 controls), confirming that all optimal matches occurred within the stringent 0.1 SD threshold. We therefore selected the 0.1 SD calliper for our final analysis, demonstrating high matching quality.

Of 1976 participants (267 CA, 1709 controls), 730 were excluded for missing NT‐proBNP. After excluding one CA patient outside the common support region, 348 participants were successfully matched (234 CA, 114 controls). The 2:1 ratio reflects the algorithm's allowance for multiple CA patients to be matched to the same control when propensity scores are highly similar within the calliper.

Balance was excellent: four of five variables achieved standardized mean difference (SMD) <0.1, with log NT‐proBNP marginally exceeding (SMD = 0.117) but representing 82.6% bias reduction. Overall metrics confirmed successful matching: mean bias 4.7%, pseudo‐*R* ^2^ reduced 97.5%, likelihood ratio test *p* = 0.667 (Table S2).

### 2.6 Outcome measures

The primary endpoint of this study was all‐cause mortality between 2012 and 2025. To ascertain events, we used follow‐up visits at our dedicated CA outpatient clinic (routinely scheduled every 6 months) and, in case of patient immobility, relied on clinical records or phone calls. Additionally, data collection on mortality was supported by regular queries to the national statistics authority's death registry, ‘Statistik Austria’.

### 2.7 Statistical analyses

Continuous variables are presented as mean ± standard deviation (SD) or median (interquartile range), depending on the distribution, assessed with the Shapiro–Wilk test. Categorical variables are expressed as absolute numbers and percentages. Comparisons between two groups were performed using independent *t*‐tests or Mann–Whitney *U* tests for continuous variables and χ2 tests for categorical variables, as appropriate. For comparisons involving more than two groups, the Kruskal–Wallis test or χ2 test was used. Box plots were generated to visualize native thoracic T1 time and ECV values across groups. The intraclass correlation coefficient (ICC) was calculated using a two‐way random‐effects model with absolute agreement and a single rater [ICC(2,1)], with 95% confidence intervals (CI) derived using Fisher's Z transformation.

Linear and logistic regression models were applied to evaluate associations between native thoracic skeletal muscle T1 time and clinical variables, both unadjusted and age‐ and sex‐adjusted. Receiver operating characteristic (ROC) curves were constructed to assess the diagnostic performance of native thoracic skeletal muscle T1 time and ECV in distinguishing CA from controls, with the area under the curve (AUC) and 95% CI calculated. The optimal diagnostic threshold was determined using the Youden index and sensitivity and specificity were calculated.

Univariate Cox regression analysis was performed to identify variables significantly associated with survival. Multivariable Cox regression analysis was subsequently conducted using a predefined model based on prior literature.^21^, ^22^ Model 1 included age, sex, body mass index and left‐ and right‐ventricular function; Model 2 additionally included native myocardial T1 time. Kaplan–Meier survival curves were constructed to assess the impact of native thoracic skeletal muscle T1 time and ECV (stratified by the median) on mortality. Differences in survival were evaluated using the log‐rank test.

All analyses were conducted using SPSS version 29.0 and Stata version 18 SE. A two‐sided *p*‐value <0.05 was considered statistically significant.

## 3 RESULTS

A total of 1976 participants were included in the study, comprising 267 patients with CA and 1709 control patients. Among CA patients, 45 patients had AL‐CA and 222 had ATTR‐CA, of whom 197 were wild‐type and 25 were hereditary. Identified TTR‐gene mutations included p.His108Arg (*n* = 9), p.Ile127Val (*n* = 3), p.Val40Ile (*n* = 2), p.Thr69Ile (*n* = 2), p.Val30Met (*n* = 2) and single cases of p.Arg5His, p.Cys30Arg, p.Phe80Leu, p.Ser97Tyr, p.Thr80Ala, p.Val113Leu and p.Val142Ile.

The control group included patients referred for evaluation of valvular heart disease (516 patients, 30.2%), heart failure (256 patients, 15.0%), left ventricular hypertrophy (203 patients, 11.9%), coronary artery disease (199 patients, 11.6%) and inflammatory conditions (130 patients, 7.6%). The remaining 405 patients (23.7%) were referred for other indications, including pericardial disease, cardiac tumours, genetic disorders and miscellaneous causes.

The mean age of the participants was 64.1 ± 17.9 years and 42.3% of the participants were female. Detailed clinical patient characteristics, including comorbidities and laboratory markers, are summarized in Table 1 for the overall cohort and separately for patients with ATTR‐ and AL‐CA.

**TABLE 1:** Baseline characteristics of controls and CA subtypes.

	Total	Controls	ATTR‐CA	AL‐CA	*p*‐Value
	*N* = 1976	*N* = 1709	*N* = 222	*N* = 45	
Age, years	64.1 (17.9)	62.2 (18.2)	78.2 (7.4)	67.6 (12.8)	**<0.001**
Female sex, %	42.26%	45.70%	16.22%	40.00%	**<0.001**
BSA, m^2^	1.91 (0.23)	1.91 (0.24)	1.92 (0.20)	1.84 (0.19)	0.38
BMI, kg/m^2^	26.77 (6.64)	26.85 (6.22)	26.47 (9.28)	24.08 (3.30)	0.14
Referral diagnosis					
VHD, %	27.18%	30.19%	9.01%	2.22%	**<0.001**
Left ventricular hypertrophy, %	21.00%	11.88%	77.93%	86.67%	
HF, %	13.51%	14.98%	4.05%	4.44%	
CAD, %	10.12%	11.64%	0.45%	0.00%	
Inflammation, %	6.63%	7.61%	0.00%	2.22%	
Others, %	21.56%	23.70%	8.56%	4.44%	
Comorbidities					
Previous myocardial infarction, %	22.04%	22.47%	17.21%	16.00%	0.31
Atrial fibrillation, %	25.76%	22.40%	45.50%	55.00%	**<0.001**
Hypertension, %	57.00%	54.36%	77.03%	60.00%	**<0.001**
Diabetes mellitus, %	15.29%	15.56%	13.96%	8.00%	0.49
Chronic obstructive pulmonary disease, %	5.12%	5.21%	4.52%	4.00%	0.88
Hyperlipidemia, %	28.53%	28.67%	28.83%	16.00%	0.38
Chronic kidney disease, %	9.75%	9.19%	11.48%	40.00%	**<0.001**
Blood parameters					
eGFR, mL/min/1.73 m^2^ (according to MDRD formula)	69.64 (29.72)	70.97 (31.34)	65.66 (19.82)	47.06 (26.72)	**<0.001**
Haematocrit, %	40.04 (5.34)	39.93 (5.48)	41.14 (4.03)	38.59 (4.66)	**0.001**
Platelet count, g/L	228.11 (83.09)	231.91 (83.75)	203.38 (65.86)	259.64 (133.87)	**<0.001**
INR	1.20 (0.42)	1.19 (0.42)	1.28 (0.43)	1.10 (0.16)	0.14
Bilirubin, mg/dL	0.69 (0.55)	0.66 (0.55)	0.84 (0.47)	0.79 (0.80)	**<0.001**
Albumin, g/L	41.39 (4.99)	41.25 (4.98)	42.54 (4.56)	38.85 (7.03)	**<0.001**
Cholinesterase, U/L	8.38 (16.06)	8.52 (16.63)	6.23 (1.70)	11.62 (21.46)	0.29
AP, U/L	79.35 (43.92)	78.18 (38.89)	82.17 (58.03)	111.65 (89.60)	**0.002**
AST, U/L	29.78 (23.45)	29.59 (25.08)	30.79 (14.29)	29.15 (8.71)	0.78
ALT, U/L	28.80 (29.07)	29.37 (31.51)	26.36 (13.44)	25.40 (11.81)	0.33
gGT, U/L	66.59 (123.91)	58.28 (80.15)	90.32 (106.48)	263.64 (675.26)	**<0.001**
NT‐proBNP, pg/mL	2936.12 (5726.43)	2697.80 (5647.66)	3209.94 (3819.92)	12423.91 (14417.71)	**<0.001**
TnT, ng/L	119.76 (466.70)	154.04 (574.81)	52.17 (34.26)	96.06 (82.95)	0.054
CK‐MB, U/L	37.75 (44.70)	38.26 (45.73)	24.08 (14.83)	37.30 (27.91)	0.59
HbA1c, %	5.84 (0.83)	5.81 (0.84)	5.96 (0.80)	5.81 (0.50)	0.091
Total cholesterol, mg/dL	163.51 (50.45)	163.10 (46.97)	152.80 (42.33)	222.95 (139.50)	**<0.001**
LDL, mg/dL	89.33 (38.64)	90.21 (38.87)	80.39 (34.57)	95.67 (45.34)	0.086
HDL, mg/dL	52.56 (17.46)	51.95 (17.86)	55.78 (14.45)	52.31 (21.77)	**0.036**
Lipoprotein(a), mg/dL	62.93 (89.75)	64.44 (92.41)	46.91 (59.17)	78.80 (75.68)	0.37
Leukocytes, G/L	7.30 (2.49)	7.35 (2.58)	7.02 (1.89)	7.52 (3.30)	0.19
Haemoglobin, g/dL	12.96 (2.02)	12.81 (2.06)	13.74 (1.61)	12.23 (1.69)	**<0.001**
CRP, mg/dL	0.95 (2.00)	1.02 (2.11)	0.54 (1.08)	0.31 (0.36)	**0.001**
Concomitant medication					
Beta‐blocker, %	57.39%	58.49%	52.76%	62.50%	0.33
ACE‐inhibitors, %	27.12%	29.44%	18.27%	14.29%	**0.005**
ARBs, %	24.12%	23.99%	24.24%	33.33%	0.81
Calcium channel blockers, %	16.63%	17.56%	12.63%	25.00%	0.20
Statin, %	38.82%	37.09%	45.81%	33.33%	0.071
T‐ASS, %	32.57%	34.47%	24.62%	42.86%	**0.025**
Coumarin, %	15.42%	14.90%	27.59%	33.33%	0.12
NOAC, %	18.09%	16.50%	47.22%	60.00%	**<0.001**
Diuretics, %	39.38%	37.01%	47.74%	71.43%	**0.005**
Tafamidis, %	9.56%	0%	83.33%	8.89%	**<0.001**
Inotersen, %	0.30%	0%	2.70%	0.00%	**<0.001**
Vutrisiran, %	0.30%	0%	2.70%	0.00%	**<0.001**
Patisiran, %	0.20%	0%	1.80%	0.00%	**<0.001**

*Note*: Bold values indicate *p* < 0.05.

Abbreviations: ACE, angiotensin‐converting enzyme; ALT, alanine aminotransferase; AP, alkaline phosphatase; ARB, angiotensin receptor blocker; AST, aspartate aminotransferase; BMI, body mass index; BSA, body surface area; CAD, coronary artery disease; CK‐MB, creatine kinase myocardial band; CMR, cardiac magnetic resonance; CRP, C‐reactive protein; eGFR, estimated glomerular filtration rate; gGT, gamma‐glutamyl transferase; HbA1c, glycated haemoglobin; HF, heart failure; INR, international normalized ratio; LA, left atrium; LDL, low‐density lipoprotein cholesterol; LVCO, left ventricular cardiac output; LVEDV, left ventricular end‐diastolic volume; LVEF, left ventricular ejection fraction; LVESV, left ventricular end‐systolic volume; LVSV, left ventricular stroke volume; NOAC, non‐vitamin K oral anticoagulants; NT‐proBNP, N‐terminal prohormone of brain natriuretic peptide; RA, right atrium; RVCO, right ventricular cardiac output; RVEDV, right ventricular end‐diastolic volume; RVEF, right ventricular ejection fraction; RVESV, right ventricular end‐systolic volume; sPAP, estimated systolic pulmonary artery pressure; T‐ASS, thrombo‐embolic prophylaxis (acetylsalicylic acid); TnT, troponin T; VHD, valvular heart disease.

Interobserver reproducibility for native thoracic skeletal muscle T1‐time was excellent (ICC [2, 1] 0.90, 95% CI 0.86–0.92, *n* = 165). The mean bias between readers was −8.7 ms, with 95% limits of agreement ranging from −93.0 to 75.6 ms. Mean absolute difference was 28.6 ms and the within‐subject coefficient of variation was 3.5%.

### 3.1 Association between CA and comorbidities

Patients with CA were significantly older than non‐CA controls (ATTR‐CA 78.2 years; AL‐CA 67.6 years vs. controls 62.2 years, *p* < 0.001) and had a higher prevalence of chronic kidney disease (*p* < 0.001, especially AL‐CA), and atrial fibrillation (*p* < 0.001). They also exhibited higher NT‐proBNP levels (*p* < 0.001) and higher gamma‐glutamyltransferase levels (*p* < 0.001) compared to the control group.

### 3.2 Comparison of CMR findings in CA versus controls

**FIGURE 2:**
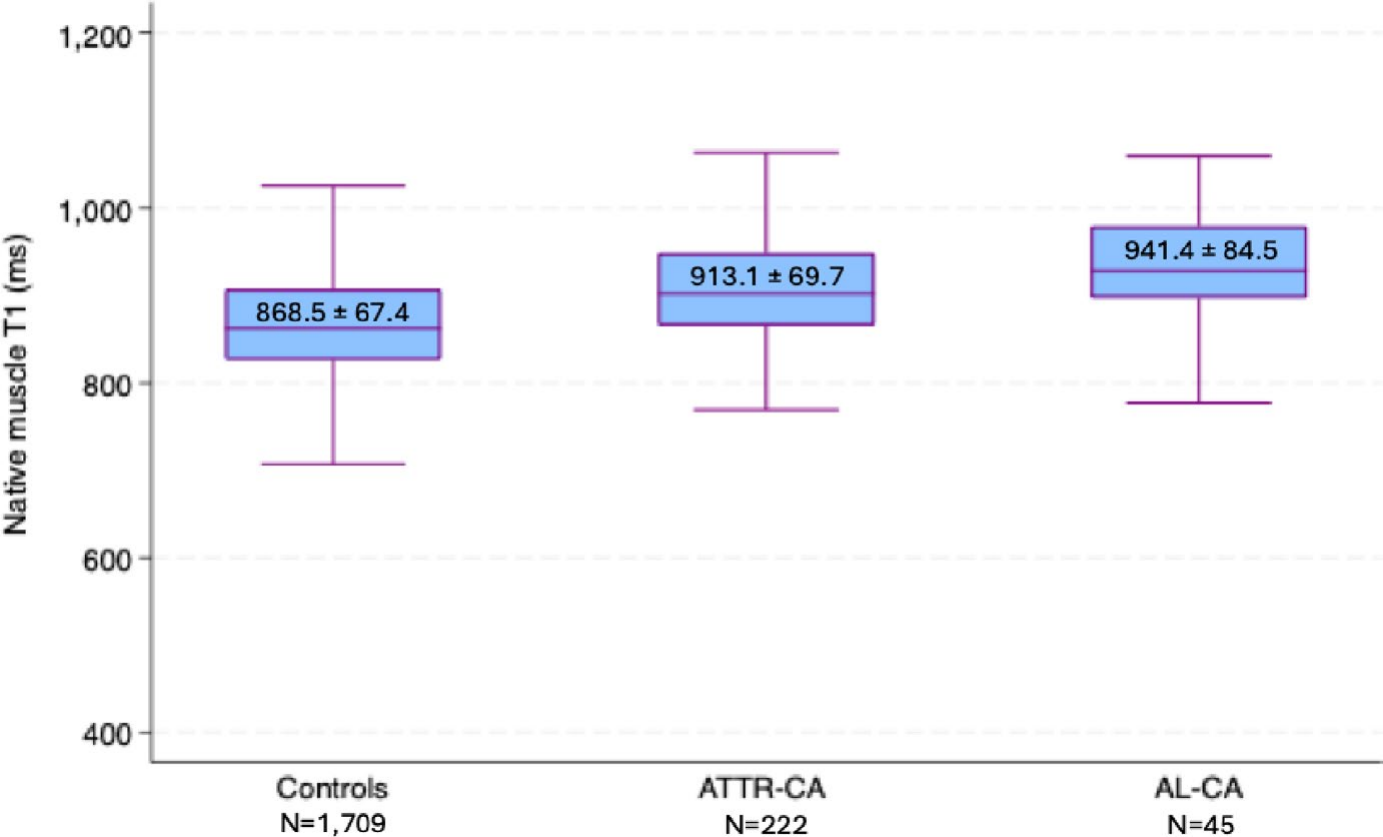
Boxplots of median native thoracic skeletal muscle T1 time in controls, ATTR‐CA and AL‐CA patients. ATTR‐CA indicates transthyretin cardiac amyloidosis; AL‐CA indicates light chain cardiac amyloidosis.

**FIGURE 3:**
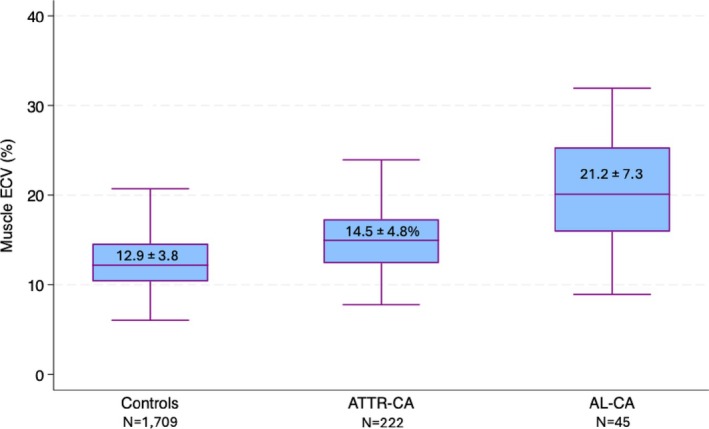
Boxplots of median skeletal muscle ECV in controls, ATTR‐CA and AL‐CA patients. ATTR‐CA indicates transthyretin cardiac amyloidosis; AL‐CA, light chain cardiac amyloidosis; ECV, extracellular volume.

Analysis of CMR imaging variables revealed significantly higher native myocardial T1 time in CA compared to controls (ATTR‐CA: 1096.3 ± 62.8 ms and AL‐CA: 1122.0 ± 68.9 ms vs. controls: 1012.3 ± 48.7 ms, *p* < 0.001). Myocardial ECV was also significantly higher in CA patients compared to controls (ATTR‐CA: 47.9 ± 14.0% and AL‐CA: 48.5 ± 15.1% vs. controls: 27.0 ± 5.0%, *p* < 0.001). Additionally, CA patients demonstrated significantly higher native thoracic skeletal muscle T1 time compared to controls (ATTR‐CA: 913.1 ± 69.7 ms and AL‐CA: 941.4 ± 84.5 ms vs. non‐CA controls: 868.5 ± 67.4 ms, *p* < 0.001) (Figure 2), accompanied by higher skeletal muscle ECV (ATTR‐CA: 14.5 ± 4.8% and AL‐CA: 21.2 ± 7.3% vs. non‐CA controls: 12.9 ± 3.8%, *p* < 0.001) (Figure 3). Analysis of inter‐observer variability demonstrated excellent agreement among readers for native thoracic skeletal muscle T1 time measurements, with an intraclass correlation coefficient of *r* = 0.90 (95% CI: 0.09–1.00, *p* = 0.021).

Correlation analysis in CA patients revealed weak but significant positive associations between myocardial and skeletal muscle tissue characterization parameters. Native myocardial T1 time correlated with native thoracic skeletal muscle T1 time (overall: *r* = 0.18, *p* = 0.034, *n* = 145), with notable differences between subtypes: ATTR‐CA showed no significant correlation (*r* = 0.05, *p* = 0.558, *n* = 121), while AL‐CA demonstrated moderate correlation (*r* = 0.52, *p* = 0.010, *n* = 24). Myocardial native T1 showed stronger correlation with thoracic skeletal muscle ECV (*r* = 0.27, *p* < 0.001, *n* = 139), while myocardial ECV correlated with skeletal muscle ECV (*r* = 0.20, *p* = 0.001, *n* = 257), with each 1% increase in myocardial ECV associated with a 0.09% increase in skeletal muscle ECV. These weak‐to‐moderate correlations (overall *R* ^2^ = 0.03–0.07, stronger in AL‐CA *R* ^2^ = 0.27) suggest that while myocardial and skeletal muscle amyloid involvement tend to occur in parallel in AL‐CA, they may progress more independently in ATTR‐CA (Figures S2 and S3).

Beyond mapping parameters, CA patients displayed significantly reduced left‐ (ATTR‐CA: 50%, AL‐CA: 54% vs. non‐CA controls: 58%) and right ventricular ejection fractions (ATTR‐CA: 47%, AL‐CA: 47% vs. non‐CA controls: 54%), larger right ventricular end‐diastolic volume indices (ATTR‐CA: 93 mL/m^2^, AL‐CA: 88 mL/m^2^ vs. non‐CA controls: 83 mL/m^2^) and increased interventricular septal thickness (ATTR‐CA: 19 mm, AL‐CA: 17 mm vs. non‐CA controls: 12 mm) than non‐CA controls. Furthermore, patients with CA showed significantly higher ventricular masses than the control group (ATTR‐CA: 184 g, AL‐CA: 181 g vs. non‐CA controls: 138 g). Detailed CMR findings for controls and CA subtypes are presented in Table 2 and Table S1.

**TABLE 2:** Baseline CMR characteristics of controls and CA subtypes.

	Total	Controls	ATTR‐CA	AL‐CA	*p*‐Value
	*N* = 1976	*N* = 1709	*N* = 222	*N* = 45	
CMR parameters					
LV mass, g	146.14 (57.08)	137.88 (54.01)	183.64 (55.49)	180.72 (60.57)	**<0.001**
LVEDV, mL	163.77 (60.29)	163.52 (62.72)	167.12 (44.92)	142.08 (26.57)	0.21
LVEDV/BSA, mL/m^2^	85.32 (29.44)	85.37 (30.00)	85.94 (23.68)	77.88 (12.86)	0.57
LVEF, %	56.90 (13.50)	58.08 (13.47)	50.13 (11.81)	53.54 (10.77)	**<0.001**
LVSV, mL	89.02 (28.77)	90.32 (29.27)	82.29 (24.98)	74.56 (17.69)	**<0.001**
LVSV/BSA, mL/m^2^	46.68 (13.72)	47.21 (13.77)	41.00 (12.15)	40.85 (8.96)	**<0.001**
IVS, mm	12.55 (4.15)	11.60 (3.25)	18.73 (4.13)	17.22 (4.56)	**<0.001**
LVCO, mL	5.81 (3.45)	5.77 (2.00)	6.17 (7.81)	4.74 (1.30)	0.12
LVCO/BSA, mL/m^2^	3.02 (1.73)	3.02 (0.96)	3.17 (5.42)	2.56 (0.71)	0.36
RVEDV, mL	160.52 (54.58)	158.19 (54.99)	174.77 (51.06)	159.87 (39.55)	**<0.001**
RVEDV/BSA, mL/m^2^	83.53 (26.61)	82.71 (26.49)	92.98 (27.27)	88.34 (20.38)	**<0.001**
RVEF, %	52.57 (10.96)	53.59 (10.59)	46.83 (11.15)	47.23 (13.06)	**<0.001**
RVSV, mL	82.01 (26.97)	82.43 (27.35)	80.24 (25.11)	72.28 (16.56)	0.16
RVSV/BSA, mL/m^2^	42.81 (13.07)	43.12 (13.11)	39.43 (12.70)	39.64 (8.40)	**0.012**
RVCO, mL	5.23 (2.36)	5.19 (1.81)	5.56 (4.37)	4.04 (1.32)	**0.012**
RVCO/BSA, mL/m^2^	2.71 (1.14)	2.71 (0.87)	2.79 (2.81)	2.18 (0.75)	0.13
LA volume, mL	37.76 (6.98)	37.67 (7.12)	38.67 (5.32)	38.97 (4.98)	0.27
LA volume/BSA, mL/m^2^	19.98 (4.15)	19.92 (4.24)	20.45 (3.23)	21.59 (2.44)	0.12
RA volume, mL	35.28 (8.29)	34.84 (6.30)	39.76 (6.87)	55.63 (52.99)	**<0.001**
RA volume/BSA, mL/m^2^	18.46 (4.74)	18.17 (3.36)	20.84 (3.63)	30.20 (29.71)	**<0.001**
Myocardial native T1, ms	1024.27 (59.35)	1012.34 (48.71)	1096.45 (62.83)	1122.96 (68.92)	**<0.001**
Blood native T1, ms	1610.05 (123.18)	1609.13 (124.67)	1602.82 (108.36)	1680.56 (115.44)	**<0.001**
Myocardial ECV, %	28.57 (8.26)	27.00 (4.96)	47.88 (13.97)	48.46 (15.08)	**<0.001**
Muscle native T1, ms	875.21 (70.23)	868.53 (67.41)	913.14 (69.74)	941.40 (84.50)	**<0.001**
Muscle ECV, %	13.55 (4.38)	12.94 (3.79)	14.50 (4.75)	21.20 (7.31)	**<0.001**

*Note*: Bold values indicate *p* < 0.05.

Abbreviations: ALT, alanine aminotransferase; AP, alkaline phosphatase; AST, aspartate aminotransferase; BMI, body mass index; BSA indicates body surface area; CA, cardiac amyloidosis; CMR, cardiac magnetic resonance; ECV, extracellular volume; gGT, gamma‐glutamyl transferase; HbA1C, glycated haemoglobin; IVS, interventricular septum; LA, left atrium; LV, left ventricle; LVCO, left ventricular cardiac output; LVEDV, left ventricular end‐diastolic volume; LVEF, left ventricular ejection fraction; LVSV, left ventricular stroke volume; NT‐proBNP, N‐terminal prohormone of brain natriuretic peptide; RA, right atrium; RV, right ventricle; RVCO, right ventricular cardiac output; RVEDV, right ventricular end‐diastolic volume; RVEF, right ventricular ejection fraction; RVSV, right ventricular stroke volume.

### 3.3 Association between native thoracic skeletal muscle T1 time and CMR parameters

Regression analysis (Table 3) demonstrated that native thoracic skeletal muscle T1 time were significantly associated with left‐ and right ventricular systolic function on CMR (LVEF: adj. beta = −0.87, *p* < 0.001; RVEF: adj. beta = −0.85, *p* < 0.001) in age and sex‐adjusted analyses. Significant correlations were also observed with ventricular size (indexed LVEDV: adj. *β* = 0.37, *p* < 0.001; indexed RVEDV: adj. *β* = 0.51, *p* < 0.001) and atrial volumes (indexed LA volume: adj. *β* = 1.98, *p* < 0.001; indexed RA volume: adj. *β* = 1.72, *p* < 0.001). Moreover, native thoracic skeletal muscle T1 time were strongly correlated with native myocardial T1 time and ECV of both the myocardium and skeletal muscle (all *p* < 0.001).

**TABLE 3:** Linear and logistic regression analysis showing associations between native thoracic skeletal muscle T1 time and clinical parameters in the overall cohort (unadjusted and age‐ and sex‐adjusted models).

Native skeletal muscle T1 time	Coef.	Linear regression			Coef.	Age‐ and sex‐adjusted		
Parameter		95% CI		*p*‐Value		95% CI		*p*‐Value
Age, years	1.084	.917	1.25	**<0.001**	1.077	.911	1.243	**<0.001**
BSA, m^2^	−57.799	−71.905	−43.694	**<0.001**	−53.948	−69.207	−38.69	**<0.001**
Serum parameters								
eGFR, mL/min/1.73 m^2^	−.36	−.485	−.235	**<0.001**	−.143	−.276	−.011	**0.034**
Haematocrit, %	−3.411	−3.972	−2.849	**<0.001**	−2.506	−3.086	−1.926	**<0.001**
Platelet count, g/L	−.007	−.051	.037	.766	.02	−.024	.064	.369
Leukocytes, G/L	−.258	−1.774	1.258	.738	−.138	−1.605	1.328	.853
CRP, mg/L	−1.321	−3.058	.415	.136	−1.182	−2.855	.491	.166
IL‐6, pg/mL	.023	−.046	.093	.509	.027	−.043	.098	.450
Haemoglobin g/dL	−7.807	−9.624	−5.99	**<0.001**	−6.451	−8.318	−4.585	**<0.001**
INR	−7.188	−17.625	3.249	.177	−9.531	−19.835	.774	.07
Bilirubin, mg/dL	1.496	−5.293	8.285	.666	2.506	−4.186	9.198	.463
Albumin, g/L	−1.746	−2.447	−1.046	**<0.001**	−1.008	−1.697	−.32	.**004**
AP, U/L	.204	.121	.288	**<0.001**	.195	.114	.276	**<0.001**
CK, U/L	−.061	−.317	.195	.633	−.025	−.307	.256	.855
CK MB, U/L	−.097	−.263	.069	.252	−.081	−.243	.081	.324
TnT, ng/L	−.001	−.013	.011	.862	0	−.012	.011	.975
Serum NT‐proBNP, pg/mL	.003	.002	.003	**<0.001**	.002	.002	.003	**<0.001**
HbA1C, %	2.524	−2.82	7.867	.354	−1.889	−7.216	3.439	.487
Imaging parameter								
LVEF, %	−.933	−1.175	−.69	**<0.001**	−.868	−1.11	−.626	**<0.001**
RVEF, %	−1.037	−1.335	−.74	**<0.001**	−.85	−1.145	−.555	**<0.001**
LVEDV/BSA, mL/m^2^	.244	.128	.36	**<0.001**	.365	.249	.481	**<0.001**
RVEDV/BSA, mL/m^2^	.404	.277	.532	**<0.001**	.512	.387	.637	**<0.001**
LA volume/BSA, mL/m^2^	3	2.173	3.827	**<0.001**	1.983	1.148	2.819	**<0.001**
RA volume/BSA, mL/m^2^	2.092	1.134	3.051	**<0.001**	1.719	.764	2.674	**0.001**
Myocardial ECV, %	2.261	1.794	2.728	**<0.001**	2.097	1.633	2.561	**<0.001**
Myocardial native T1, ms	.432	.384	.481	**<0.001**	.382	.334	.43	**<0.001**

	Odds ratio	Logistic regression			Coef.	Age, and sex‐adjusted		
		95% CI		*p*‐Value		95% CI		*p*‐Value
Sex, male, %	−11.817	−18.069	−5.564	**0.001**	−11.01	−17.023	−4.997	**0.001**
Comorbidities								
Hypertension, %	6.692	.529	12.854	.033	−6.573	−12.789	−.358	.**038**
Atrial fibrillation, %	22.618	15.443	29.793	**<0.001**	11.278	3.999	18.557	.**002**
Diabetes mellitus, %	8.548	−.016	17.111	.05	−1.334	−9.685	7.017	.754
Hyperlipidemia, %	−5.068	−11.896	1.76	.146	−15.47	−22.151	−8.79	**<0.001**
COPD, %	13.362	−.6	27.325	.061	4.434	−9.022	17.89	.518
CAD, %	13.787	6.165	21.409	**<0.001**	3.634	−4.073	11.341	.355
Chronic kidney disease, %	28.543	17.937	39.148	**<0.001**	22.915	12.632	33.198	**<0.001**
HFrEF/HFmrEF, %	28.376	20.554	36.198	**<0.001**	22.967	15.136	30.798	**<0.001**

*Note*: Bold values indicate *p* < 0.05.

Abbreviations: ALT, alanine aminotransferase; AP, alkaline phosphatase; AST, aspartate aminotransferase; BMI, body mass index; BSA, body surface area; CAD, coronary artery disease; COPD, chronic obstructive pulmonary disease; CRP, C‐reactive protein; ECV, extracellular volume; eGFR, estimated glomerular filtration rate; gGT, gamma‐glutamyltransferase; HbA1C, glycated haemoglobin; INR, International Normalized Ratio; LA, left atrium; LVEF, left ventricular ejection fraction; NT‐proBNP, N‐terminal prohormone of brain natriuretic peptide; RA, right atrium; RVEF, right ventricular ejection fraction.

A strong linear relationship was observed between native thoracic skeletal muscle T1 time and both eGFR and serum NT‐proBNP (*p* ≤ 0.034). Additional significant age‐ and sex‐adjusted associations were identified between native thoracic skeletal muscle T1 time and haematocrit, haemoglobin, albumin and alkaline phosphatase (all *p* ≤ 0.004).

### 3.4 Association between native thoracic skeletal muscle T1 times and comorbidities

Increasing native thoracic skeletal muscle T1 time was strongly associated with a higher prevalence of atrial fibrillation (OR = 11.28, *p* < 0.001) and chronic kidney disease (OR = 22.9, *p* < 0.001) after adjustment for age and sex. Native thoracic skeletal muscle T1 time was also associated with coronary artery disease (OR = 13.79, *p* < 0.001), however, this association was not maintained after adjustment for age and sex. Additionally, native thoracic skeletal muscle T1 time was significantly correlated with age, sex, and body surface area (all *p* < 0.001; Table 3).

### 3.5 ROC analysis of predictors for CA

**FIGURE 4:**
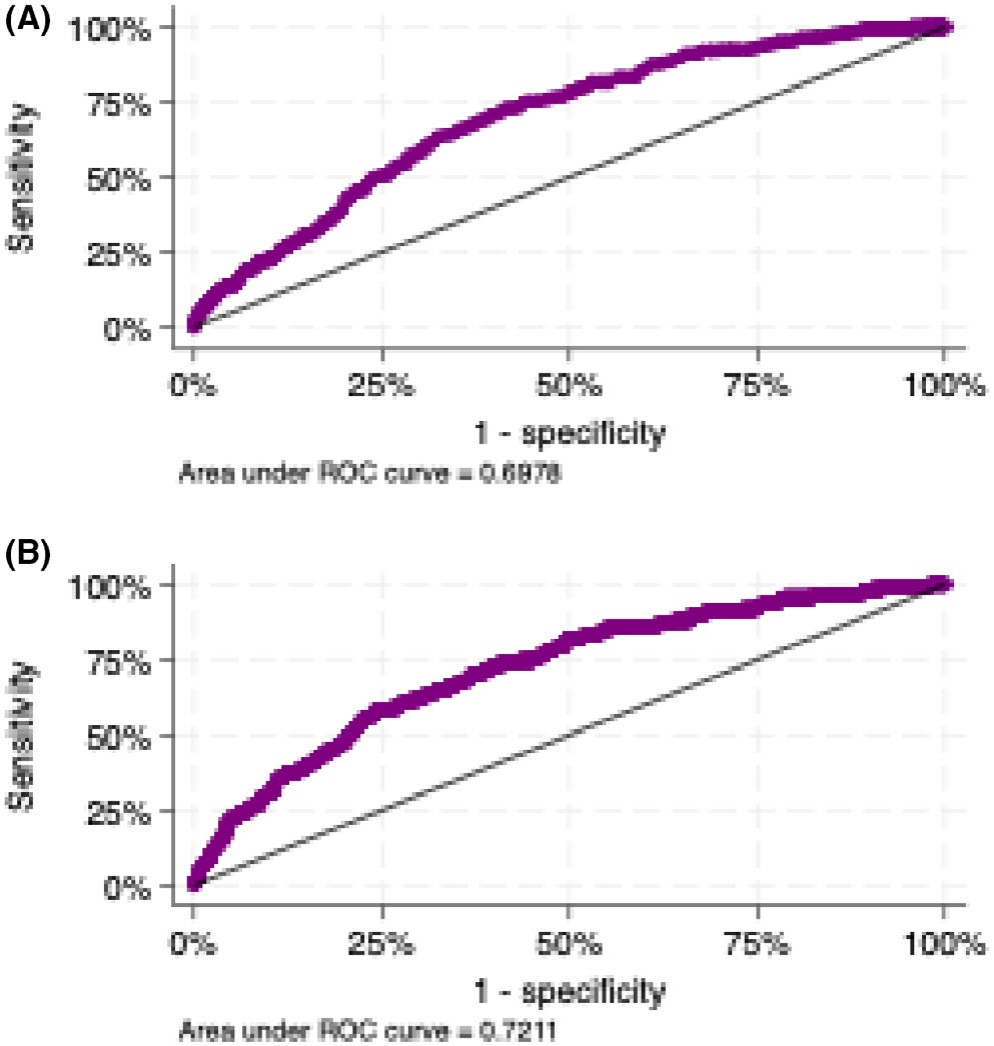
Receiver operating characteristic curves illustrating the diagnostic performance of (A) native thoracic skeletal muscle T1 times and (B) thoracic skeletal muscle extracellular volume in distinguishing patients with cardiac amyloidosis from controls.

ROC curve analysis (Figure 4) demonstrated that native skeletal muscle T1 time had moderate predictive value for distinguishing CA patients from controls, with an AUC of 0.70 (95% CI, 0.67–0.73). A native thoracic T1 time threshold of 895 ms yielded a sensitivity and specificity of 61% and 59%, respectively. Skeletal muscle ECV also showed predictive capability, with an AUC of 0.72. In clinically relevant subgroups, skeletal muscle T1 time maintained discriminatory ability, as summarized in Table S4. After adjustment for age, sex, and body surface area, the predictive performance improved, with an AUC of 0.83 (95% CI, 0.79–0.87; *p* < 0.001).

### 3.6 Association between native thoracic skeletal muscle T1 times, CA and outcome

During a median follow‐up of 5.0 years (IQR: 2.6–9.0 years), 442 deaths occurred (22.4% overall mortality). The CA group had 76 deaths (28.5% event rate; median follow‐up 2.7 years, IQR 1.0–5.1 years) compared to 366 deaths in controls (21.4% event rate; median follow‐up 5.7 years, IQR 2.8–9.5 years). Among CA subtypes, ATTR‐CA patients had 55 events (24.8%; median follow‐up 3.0 years, IQR 1.0–5.2 years) and AL‐CA patients had 21 events (46.7%; median follow‐up 1.8 years, IQR 0.4–3.4 years). Event rates per 100 person‐years were 9.0 for CA versus 3.5 for controls. CA patients had significantly worse survival (unadjusted hazard ratio 2.05, 95% CI 1.60–2.63, log‐rank *p* < 0.001).

Overall, higher native thoracic skeletal muscle T1 time was associated with an increased risk of death in univariate analysis (HR = 1.65, 95% CI [1.50–1.81], per 100 ms increase, *p* < 0.001). This association remained significant after adjustment for age, sex, left‐ and right‐ventricular function and body mass index (Model 1: HR = 1.21, 95% CI [1.05–1.39], per 100 ms increase, *p* = 0.008; left side of Table 4). However, after additional adjustment for native myocardial T1 time (Model 2), skeletal muscle T1 time was no longer independently associated with survival (HR = 1.06, 95% CI [0.91–1.24], per 100 ms increase, *p* = 0.42; right side of Table 4).

**TABLE 4:** Multivariable Cox regression analysis of the association of native thoracic skeletal muscle T1 time with all‐cause mortality.

Variable	Model 1: Adjusted clinical model			Model 2: Additionally adjusted for myocardial T1		
	HR	95% CI	*p*‐Value	HR	95% CI	*p*‐Value
Native thoracic skeletal muscle T1 time^a^	**1.210**	1.051–1.393	**0.008**	1.064	0.914–1.239	0.420
Age, years	**1.054**	1.044–1.064	**<0.001**	**1.051**	1.041–1.061	**<0.001**
Male sex	**1.253**	1.001–1.569	**0.049**	1.257	0.999–1.582	0.051
LVEF, %	0.996	0.987–1.006	0.409	0.999	0.989–1.010	0.830
RVEF, %	**0.975**	0.964–0.987	**<0.001**	**0.978**	0.967–0.989	**<0.001**
BMI, kg/m^2^	0.991	0.973–1.010	0.374	0.995	0.976–1.015	0.628
Native myocardial T1 time^a^	–	–	–	**1.603**	1.290–1.993	**<0.001**

*Note*: Model 1: Adjusted for age, sex, LVEF, RVEF and BMI. Model 2: Additionally adjusted for native myocardial T1 time. Bold values indicate statistical significance (*p* < 0.05).

Abbreviations: BMI, body mass index; CI, confidence interval; HR, hazard ratio; LVEF, left ventricular ejection fraction; RVEF, right ventricular ejection fraction.

^a^ Per 100 ms increase.

**FIGURE 5:**
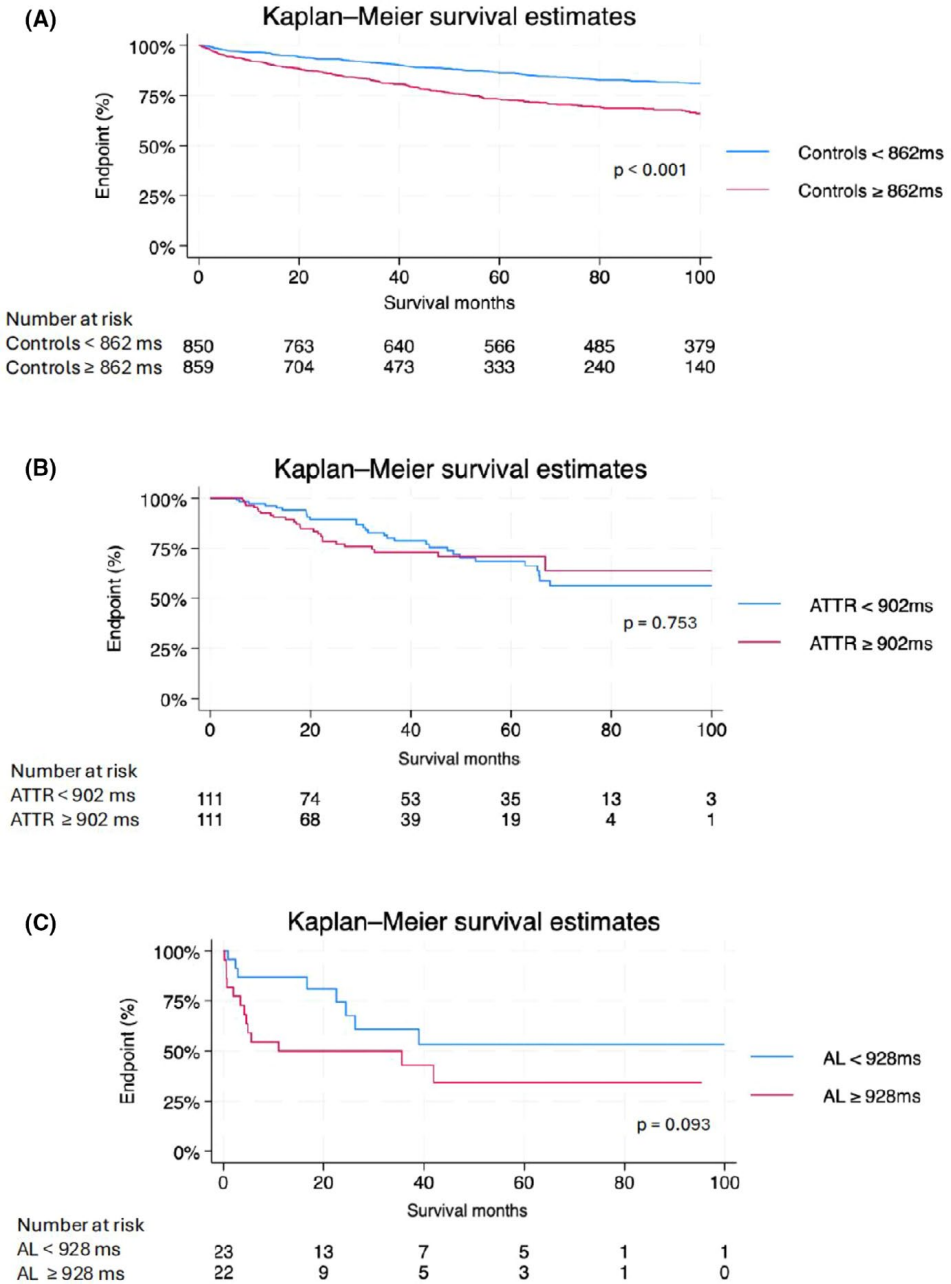
Kaplan–Meier survival curves illustrating overall survival according to native thoracic skeletal muscle T1 time stratified by the median in (A) controls, (B) ATTR‐CA patients and (C) AL‐CA patients. ATTR‐CA indicates transthyretin cardiac amyloidosis; AL‐CA, light chain cardiac amyloidosis.

**FIGURE 6:**
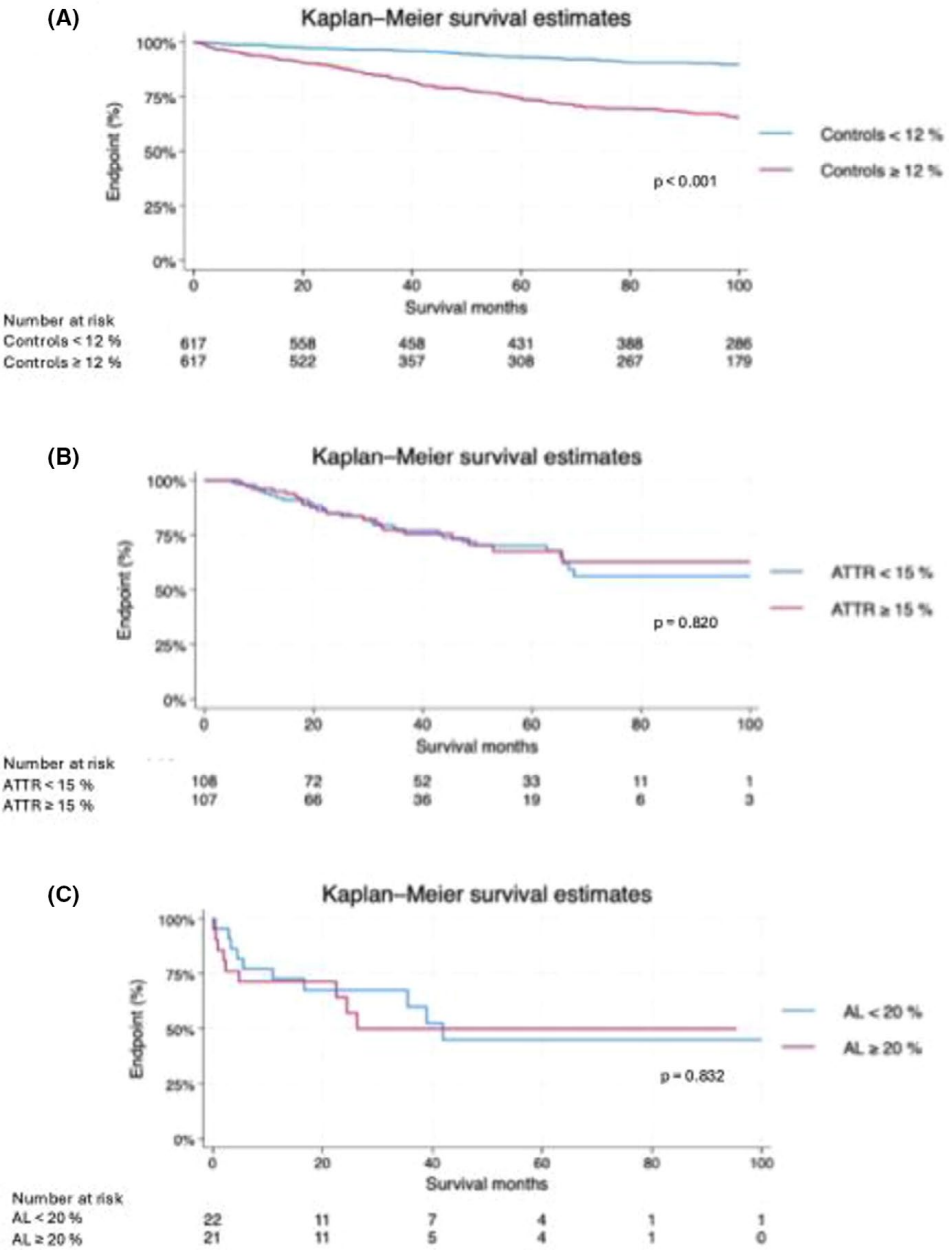
Kaplan–Meier survival curves illustrating overall survival according to ECV stratified by the median in (A) controls, (B) ATTR‐CA and (C) AL‐CA patients. ATTR‐CA indicates transthyretin cardiac amyloidosis; AL‐CA indicates light chain cardiac amyloidosis; ECV, extracellular volume.

Patients with CA had significantly worse outcomes than controls (HR = 2.15, 95% CI [1.68–2.76], *p* = 0.001). Subgroup analyses revealed that higher native thoracic skeletal muscle T1 time was associated with worse survival in controls (*p* < 0.001), but not in ATTR‐CA (*p* = 0.753) or AL‐CA (*p* = 0.093) (Figure 5). Similarly, higher skeletal muscle ECV was associated with worse survival in controls (*p* < 0.001), but showed no significant association with survival in patients with ATTR‐CA (*p* = 0.820) or AL‐CA (*p* = 0.832) (Figure 6).

### 3.7 Propensity score matching

To account for baseline imbalances between CA patients and controls, propensity score matching was performed, adjusting for age, sex, eGFR, NT‐proBNP and atrial fibrillation (Tables S2 and S3). After matching, major baseline imbalances were largely attenuated.

Importantly, myocardial tissue characterization parameters remained markedly different after matching, with significantly higher native myocardial T1 time (*p* < 0.001) and myocardial ECV (*p* < 0.001) in CA patients. Likewise, native thoracic skeletal muscle T1 time (*p* < 0.001) and thoracic skeletal muscle ECV (*p* < 0.001) remained significantly higher in CA patients than in controls, whereas native blood T1 time showed the opposite pattern (*p* < 0.001).

## 4 DISCUSSION

This study presents three key findings: (i) Patients with CA exhibit significantly higher native thoracic skeletal muscle T1 time compared to controls, (ii) skeletal muscle ECV is also markedly higher in CA patients and (iii) higher native thoracic skeletal muscle T1 time is associated with worse survival in the control group, but not in patients with ATTR‐CA or AL‐CA. These results suggest that native thoracic skeletal muscle T1 time and ECV may serve as novel, noninvasive markers of systemic amyloid deposition, providing valuable insights into extracardiac involvement in CA. The ability to obtain these measurements via CMR imaging offers a safer alternative to invasive procedures such as endomyocardial biopsy. Our finding that in non‐CA patients, native thoracic skeletal T1 time was associated with outcome warrants further studies.

### 4.1 Muscle involvement and histological evidence

Histological studies have demonstrated that amyloid deposition in skeletal muscle is prevalent in amyloidosis. In a study of 28 patients with systemic amyloidosis, 86% (24 patients) had amyloid deposits in skeletal muscle biopsies,^8^ despite only a few patients reporting symptoms of myopathy.

Additionally, radiotracer uptake in skeletal muscle has been observed on technetium bone scintigraphy,^23^, ^24^, ^25^, ^26^ particularly in patients with wild‐type ATTR‐CA and variant ATTR‐CA (Val142Ile mutation), further supporting the concept of muscular involvement in CA. One study provided histological confirmation of skeletal muscle amyloid deposits in patients with Perugini grade 3 cardiac uptake.^25^ Another study identified amyloid deposits in the internal oblique muscle via computed tomography‐guided biopsy and technetium‐labelled radiotracers, highlighting skeletal muscle as a relevant site for amyloid detection.^27^

Several reports illustrate the frequent coexistence of ATTR‐CA and ATTR‐myopathy.^27^, ^28^, ^29^ One such report^30^ described a patient with isolated amyloid myopathy in whom no amyloid deposits were found in the rectal mucosa, cardiac muscle or sural nerve, suggesting that amyloid deposits can occur solely in skeletal muscle, independent of other organs. Takahashi et al.^27^ recommend considering skeletal muscle biopsy when fat pad biopsies are negative or inconclusive. However, given the invasive nature and inherent risks of biopsy procedures, there remains an urgent need for reliable noninvasive methods to evaluate muscular involvement in amyloidosis. These observations highlight the potential value of native thoracic skeletal muscle T1 time as an adjunctive, noninvasive marker in the diagnostic workup of suspected CA, potentially improving diagnostic accuracy.

### 4.2 Skeletal muscle assessment using CMR

While CMR is widely used for noninvasive tissue characterization via T1‐mapping, primarily for the myocardium in CA patients, our study extends this application to skeletal muscles, suggesting that elevated native thoracic skeletal muscle T1 time and ECV reflect systemic amyloid deposition. This is particularly relevant in cases where myocardial involvement is subtle or difficult to detect. Unlike the myocardium, skeletal muscle is less influenced by hemodynamic fluctuations, thereby enhancing the reproducibility of T1 time measurements.

CMR T1‐mapping is considered the gold standard for noninvasive tissue characterization, allowing differentiation between focal and diffuse fibrosis in cardiac disease. It has been proposed as a noninvasive alternative to myocardial biopsy^31^, ^32^ and is widely used for diagnosis and prognostication in cardiovascular diseases.^33^, ^34^ High native T1 time has been described as a marker of diffuse microfibrosis, detecting tissue changes earlier than late gadolinium enhancement.^35^ Recent studies have expanded the use of T1‐mapping beyond the heart to other organs, such as the liver,^36^, ^37^ spleen,^38^ pancreas^38^ and renal cortex.^39^, ^40^ These investigations have shown associations between elevated native T1 times, disease pathology and adverse clinical outcomes.^39^ In amyloidosis patients, high ECV in the liver and spleen has been linked to greater amyloid infiltration, suggesting the utility of T1‐mapping in assessing visceral/systemic amyloid burden.^41^ A genome‐wide association study has further shown an association between increased organ‐specific native T1 time and various prevalent diseases across multiple organ systems.^40^

While native myocardial T1 time and ECV are often markedly elevated in CA,^42^ skeletal muscle tissue is visible on standard cardiac T1‐maps, but has not been previously quantified in CA. T1‐mapping of skeletal muscle provides superior tissue characterization without radiation exposure, offering advantages over imaging modalities such as computed tomography and dual‐energy X‐ray absorptiometry, which can quantify muscle mass or density,^43^, ^44^ but do not detect muscle fibrosis and expose patients to radiation. Additionally, while muscle biopsy is limited to small tissue samples, magnetic resonance imaging enables the assessment of larger parts of skeletal muscle, which constitutes a significant proportion of total body weight.^45^ A prior study from our group demonstrated the prognostic value of native thoracic skeletal muscle T1 time in patients with heart failure with preserved ejection fraction,^46^ further supporting its clinical relevance.

### 4.3 Diagnostic and prognostic utility of native thoracic skeletal muscle T1 time and ECV

This study is the first to quantify native thoracic skeletal muscle T1 time in CA patients and offers novel insights into its diagnostic utility. ROC curve analysis showed that native thoracic skeletal muscle T1 time exhibits moderate diagnostic performance (AUC = 0.70) for CA, suggesting it is a suitable marker for distinguishing CA from other pathologies. The proposed threshold of 895 ms showed moderate sensitivity and specificity, limiting its utility as an independent diagnostic marker and suggesting that it should primarily be applied as a complementary imaging finding to raise suspicion for CA rather than confirm the diagnosis.

Baggiano et al.^47^ reported a slightly higher AUC for native myocardial T1 time (AUC = 0.93) in differentiating CA patients from controls, suggesting that native myocardial T1 time remains the superior parameter. This is in line with our study, showing an AUC of 0.88 (95% CI, 0.85–0.90) for native myocardial T1 time and an AUC of 0.93 (95% CI, 0.92–0.96) for myocardial ECV (as shown in Figure S1). However, native thoracic skeletal muscle T1 time could serve as a complementary tool, particularly in patients with minimal or noncardiac symptoms or in early‐stage disease. Although skeletal thoracic muscle native T1 time does not provide incremental diagnostic value beyond myocardial mapping, it may serve as a complementary tool in specific scenarios, particularly in patients with minimal or noncardiac symptoms or early‐stage disease. Practical advantages include assessment without additional sequences or scan time. Skeletal muscle evaluation can provide supportive diagnostic information when myocardial assessment is compromised by pacemakers/ICDs, extensive myocardial scar, borderline myocardial ECV values, arrhythmia‐ or motion‐related image quality limitations or when corroborative evidence of extracardiac involvement is desired, without the need for dedicated skeletal muscle imaging.

As expected, native thoracic myocardial T1 time and ECV were significantly higher than skeletal muscle values in both CA patients and controls in this study, aligning with findings from previous studies.^46^, ^48^ Normal myocardial ECV values in healthy individuals are approximately 25% ± 3.5%,^42^ while skeletal muscle ECV is generally lower, around 10%.^49^ This difference is likely due to the myocardium's distinct structure, including a higher collagen content, greater perfusion and continuous activity, which could promote amyloid deposition in cardiac tissue and explain the frequent cardiac manifestations of amyloidosis.^49^ Despite this, native thoracic skeletal muscle T1 time also exhibited strong diagnostic performance in our study, indicating its potential for identifying systemic amyloid involvement.

We further observed a significant correlation between myocardial and skeletal muscle ECV, suggesting shared underlying disease mechanisms. Interestingly, high native thoracic skeletal muscle T1 time was associated with worse survival in the control group, but this association was not observed in the CA subgroups. One possible explanation for the lack of association in our CA cohort is the markedly reduced overall survival in these patients, which may overshadow more subtle prognostic effects of skeletal muscle involvement. In the AL‐CA subgroup, higher native thoracic skeletal muscle T1 time showed a trend toward poorer outcomes, although the cohort size was too small to reach statistical significance. In addition, skeletal muscle amyloid deposition is often clinically silent, whereas cardiac infiltration directly causes symptomatic limitations such as dyspnea, leading to a stronger and earlier impact on prognosis.

Musculoskeletal symptoms, such as carpal tunnel syndrome, lumbar spinal stenosis and skeletal myopathy, commonly referred to as ‘extracardiac red flags’, often precede cardiac and neuropathic symptoms by several years.^50^ In some patients, skeletal myopathy may even be the first or sole manifestation of wild‐type ATTR‐CA.^28^ Thus, skeletal muscle T1 time may serve as a valuable imaging marker for earlier disease detection and improved risk stratification in CA. While thoracic native skeletal muscle T1 time shows promise as a diagnostic marker for cardiac amyloidosis, our survival analysis did not demonstrate prognostic value within the CA population (HR 1.15 per 100 ms increase, 95% CI 0.90–1.46, *p* = 0.27). This suggests that while native thoracic skeletal muscle T1 time elevation is characteristic of CA, it may not reflect the severity or progression of myocardial involvement that drives clinical outcomes. Future studies should evaluate whether changes in native thoracic skeletal muscle T1 time during disease‐modifying therapy correlate with treatment response or clinical improvement.

To enhance clinical applicability, future studies should further investigate larger cohorts to define reference values, monitor longitudinal changes, and evaluate the prognostic implications of both high and low thoracic skeletal muscle T1 time and ECV in CA patients. Standardization of thoracic skeletal muscle T1 time thresholds across different CMR vendors will be essential for broader clinical application. Additionally, exploring other skeletal muscle groups, such as the gluteus maximus or quadriceps, could provide complementary insights into amyloid distribution patterns. Finally, external validation of our findings, ideally with histological confirmation, will be crucial to confirm the diagnostic relevance of skeletal muscle T1‐mapping in CA, especially considering that disease‐modifying treatments are most effective when initiated early.

### 4.4 Study limitations

This series represents the most extensive study to date investigating the association between native thoracic skeletal muscle T1 time and CA. Despite the large sample size, several limitations warrant consideration. First, data were collected at a single center. While this ensured consistency in CMR acquisition, reporting, patient work‐up and post‐processing workflows, thereby minimizing inter‐institutional variability, referral bias cannot be excluded. Nonetheless, this approach reflects real‐world clinical practice and enhances the applicability of our findings to routine patient care.

Second, muscle biopsies were not performed, limiting our ability to definitively attribute elevated native thoracic T1 time to amyloid deposition in skeletal muscle, as other factors, such as inflammation, fibrosis or fat infiltration, may also contribute to T1 time alterations. While native thoracic skeletal muscle T1 time may also be elevated in other myopathies, we excluded patients with known severe myopathies to reduce confounding. Systemic inflammation showed no association with native thoracic skeletal muscle T1 time, indicating that inflammatory processes did not substantially confound skeletal muscle T1 time.

Third, skeletal muscle T1‐maps were derived from a single slice, which may not fully capture amyloid deposition throughout the entire muscle. However, this approach allows assessment of larger muscle regions compared with biopsy, which samples only a small portion of tissue.

Finally, thoracic skeletal muscle was used as the sole site for T1 time measurement, potentially limiting generalizability. However, thoracic skeletal muscle is easily accessible in routine CMR protocols, making this approach clinically feasible.

Despite these limitations, our study provides novel insights into the potential utility of skeletal muscle T1‐mapping for CA diagnosis and risk stratification.

## 5 CONCLUSION

Native thoracic skeletal muscle T1 time and ECV, as measured non‐invasively via CMR, are significantly higher in CA patients than in controls, likely reflecting systemic amyloid infiltration, including the skeletal muscle. These markers expand the diagnostic utility of CMR T1‐mapping beyond the myocardium and could serve as adjunctive markers for early detection and risk stratification of CA patients.

## AUTHOR CONTRIBUTIONS

Christina Kronberger and Katharina Mascherbauer: conception, analysis and interpretation of data, manuscript drafting. Michael Poledniczek, Lena Marie Schmid, Carolina Donà, Matthias Koschutnik, Laura Lunzer, René Rettl, Franz Duca, Christina Binder, Christian Nitsche, Nikita Ermolaev and Varius Dannenberg: conception, analysis and interpretation of data, revising the manuscript. Gözde Celebi: analysis of data and manuscript drafting. Dietrich Beitzke: analysis and interpretation of data, revising the manuscript and supervision. Roza Badr Eslam: conception and analysis, revising the manuscript and supervision. Jutta Bergler‐Klein, Julia Mascherbauer, Johannes Kastner, Christian Hengstenberg and Andreas Anselm Kammerlander: conception, analysis and interpretation of data, revising the manuscript and supervision. All authors approved the manuscript for publication.

## CONFLICT OF INTEREST STATEMENT

The authors declare no conflicts of interest.

## Supporting information


Appendix S1.

## Data Availability

The data that support the findings of this study are available on request from the corresponding author. The data are not publicly available due to privacy or ethical restrictions.
