# Supplementary material for: CMR‐derived skeletal muscle T1 time and extracellular volume as novel diagnostic markers for cardiac amyloidosis
Source: Eur J Clin Invest. 2026 Mar 30;56(4):e70191. doi: 10.1111/eci.70191 (PMC13034890; doi:10.1111/eci.70191)
Supplement: Supplementary file 1 — Appendix S1. [file ECI-56-e70191-s001.docx]

**Supplementary material**

******

**Supplementary Figure 1.** Receiver operating characteristic curves illustrating the diagnostic performance of **(A)** native myocardial T1 times and **(B)** myocardial extracellular volume in distinguishing patients with cardiac amyloidosis from controls. ROC indicates receiver operating characteristic.

**Supplementary Figure 2.** Correlations between myocardial and skeletal muscle tissue characterization parameters in cardiac amyloidosis patients: **(A)** Muscle T1 vs myocardial T1 **(B)** Muscle ECV vs myocardial ECV **(C)** Muscle T1 vs myocardial ECV **(D)** Muscle ECV vs myocardial T1. Red lines indicate linear regression fits. **ECV indicates extracellular volume; T1, T1 time.**

**Supplementary Figure 3.** **Myocardial-skeletal muscle T1 correlation by CA subtype.** Differential correlation patterns were observed between subtypes. ATTR-CA (blue circles, n = 121) showed no correlation (r = 0.05, p = 0.558, flat blue line), whereas AL-CA (red triangles, n = 24) demonstrated moderate correlation (r = 0.52, p = 0.010, dashed red line). Overall: r = 0.18, p = 0.034, n = 145. Horizontal line: skeletal muscle T1 cut-off 895 ms. Findings suggest parallel myocardial-skeletal muscle progression in AL-CA versus independent progression in ATTR-CA, reflecting distinct pathophysiology between subtypes. AL-CA indicates light-chain cardiac amyloidosis; ATTR-CA, transthyretin cardiac amyloidosis.

**Supplementary Table 1.** CMR mapping parameters of controls and CA subtypes

|  | **Total** | **Controls** | **ATTR-CA** | **AL-CA** | **p-value** |  |
| --- | --- | --- | --- | --- | --- | --- |
|  | N=2031 | N=1709 | N=238 | N=84 |  |  |
| **T1 time myocardium, ms** | | | | | |  |
| Pre-contrast agent | 1024.27 (59.35) | 1012.34 (48.71) | 1096.45 (62.83) | 1120.96 (68.92) | **<0.001** |  |
| Post-contrast agent | 430.18 (60.17) | 442.39 (47.84) | 345.89 (70.25) | 404.34 (66.12) | **<0.001** |  |
| **T1 time skeletal muscle, ms** | | | | | |  |
| Pre-contrast agent | 875.21 (70.23) | 868.53 (67.41) | 913.14 (69.74) | 941.40 (84.50) | **<0.001** |  |
| Post-contrast agent | 564.15 (47.26) | 564.12 (47.57) | 566.92 (45.06) | 551.07 (48.04) | 0.13 |  |
| **T1 time blood, ms** | | | | | |  |
| Pre-contrast agent | 1610.05 (123.18) | 1609.13 (124.67) | 1602.82 (108.36) | 1680.56 (115.44) | **<0.001** |  |
| Post-contrast agent | 295.34 (53.83) | 291.25 (52.60) | 313.89 (50.09) | 351.61 (64.94) | **<0.001** |  |
| **ECV, %** | | | | | | |
| Myocardial | | 28.57 (8.26) | 27.00 (4.96) | 47.88 (13.97) | 48.46 (15.08) | **<0.001** |
| Skeletal muscle | | 13.55 (4.38) | 12.94 (3.79) | 15.50 (4.75) | 21.20 (7.31) | **<0.001** |

CMR indicates cardiac magnetic resonance; CA, cardiac amyloidosis; ATTR-CA, transthyretin cardiac amyloidosis; AL-CA, light-chain cardiac amyloidosis; ECV, extracellular volume; ms, milliseconds. Bold values indicate p <0.05.

**Supplementary Table 2.** Baseline Characteristics After Propensity Score Matching (Caliper 0.1 SD).

|  | **Total** | | **Controls** | | **CA** | **ATTR-CA** | | | **AL-CA** | **p-value** | |
| --- | --- | --- | --- | --- | --- | --- | --- | --- | --- | --- | --- |
|  | N=470 | | N=235 | | N=235 | N=217 | | | N=18 |  | |
| Age, years | 77.6 (8.2) | | 77.2 (8.5) | | 77.9 (7.9) | 78.3 (7.5) | | | 73.2 (10.5) | **0.029** | |
| Female sex, % | 17.23% | | 16.60% | | 17.87% | 15.67% | | | 44.44% | **0.008** | |
| BSA, m^2^ | 1.93 (0.20) | | 1.95 (0.21) | | 1.91 (0.20) | 1.92 (0.20) | | | 1.84 (0.20) | 0.058 | |
| BMI, kg/m^2^ | 26.53 (7.19) | | 26.68 (4.47) | | 26.37 (9.13) | 26.50 (9.38) | | | 24.56 (3.58) | 0.54 | |
| **Referral diagnosis** |  | |  | |  |  | | |  | **<0.001** | |
| VHD, % | 33.19% | | 57.87% | | 8.51% | 8.76% | | | 5.56% |  | |
| Left ventricular hypertrophy, % | 44.68% | | 11.49% | | 77.87% | 78.34% | | | 72.22% |  | |
| HF, % | 7.02% | | 9.79% | | 4.26% | 4.15% | | | 5.56% |  | |
| CAD, % | 3.19% | | 5.96% | | 0.43% | 0.46% | | | 0.00% |  | |
| Inflammation, % | 0.85% | | 1.28% | | 0.43% | 0.00% | | | 5.56% |  | |
| Others, % | 11.06% | | 13.62% | | 8.51% | 8.29% | | | 11.11% |  | |
| **Comorbidities** |  | |  | |  |  | | |  |  | |
| Previous myocardial infarction, % | 35.85% | | 45.96% | | 18.38% | 17.80% | | | 22.22% | **<0.001** | |
| Atrial fibrillation, % | 44.26% | | 41.70% | | 46.81% | 45.62% | | | 61.11% | 0.24 | |
| Hypertension, % | 60.64% | | 70.21% | | 51.06% | 50.23% | | | 61.11% | **<0.001** | |
| Diabetes mellitus, % | 21.28% | | 28.51% | | 14.04% | 14.29% | | | 11.11% | **<0.001** | |
| Chronic obstructive pulmonary disease, % | 7.25% | | 9.79% | | 4.70% | 4.52% | | | 4.00% | 0.88 | |
| Hyperlipidemia, % | 34.47% | | 40.43% | | 28.51% | 29.03% | | | 22.22% | **0.021** | |
| Chronic kidney disease, % | 18.60% | | 19.15% | | 17.65% | 11.86% | | | 55.56% | **<0.001** | |
| **Blood parameters** |  | |  | |  |  | | |  |  | |
| eGFR, ml/min/1.73 m^2^ (according to MDRD formula) | 64.74 (25.24) | 65.15 (28.90) | | 64.34 (21.00) | | | 65.72 (19.84) | 47.60 (27.39) | | | **0.013** |
| Hematocrit, % | 39.14 (5.23) | 37.48 (5.59) | | 40.79 (4.25) | | | 41.17 (4.00) | 36.23 (4.69) | | | **<0.001** |
| Platelet count, g/L | 205.10 (68.43) | 205.12 (68.30) | | 205.09 (68.70) | | | 203.18 (66.13) | 228.06 (93.76) | | | 0.33 |
| INR | 1.27 (0.51) | 1.28 (0.55) | | 1.25 (0.41) | | | 1.28 (0.43) | 1.07 (0.15) | | | 0.32 |
| Bilirubin, mg/dL | 0.78 (0.51) | 0.74 (0.51) | | 0.83 (0.50) | | | 0.83 (0.47) | 0.77 (0.82) | | | 0.16 |
| Albumin, g/L | 41.24 (4.93) | 40.17 (4.80) | | 42.28 (4.85) | | | 42.52 (4.59) | 39.47 (6.77) | | | **<0.001** |
| Cholinesterase, U/L | 6.22 (1.91) | 6.18 (2.00) | | 6.31 (1.71) | | | 6.23 (1.70) | 6.72 (1.74) | | | 0.54 |
| AP, U/L | 83.07 (49.80) | 82.25 (36.11) | | 83.90 (60.55) | | | 81.96 (58.01) | 106.94 (83.92) | | | 0.12 |
| AST, U/L | 28.88 (14.83) | 27.07 (15.43) | | 30.68 (14.01) | | | 30.81 (14.37) | 29.06 (8.79) | | | 0.028 |
| ALT, U/L | 25.81 (18.81) | 25.44 (23.09) | | 26.17 (13.29) | | | 26.36 (13.48) | 23.94 (10.85) | | | 0.80 |
| gGT, U/L | 77.40 (93.65) | 64.84 (79.43) | | 89.96 (104.66) | | | 90.47 (106.89) | 83.83 (74.91) | | | **0.014** |
| NT-proBNP, pg/mL | 3833.18 (5694.47) | 3840.77 (5766.40) | | 3825.60 (5633.92) | | | 3216.41 (3832.35) | 11169.68 (13727.76) | | | **<0.001** |
| TnT, ng/L | 77.40 (93.65) | 64.84 (79.43) | | 89.96 (104.66) | | | 52.18 (34.35) | 96.80 (85.81) | | | **0.028** |
| CK-MB, U/L | 34.07 (43.52) | 35.08 (47.68) | | 30.25 (22.14) | | | 24.08 (14.83) | 39.94 (29.04) | | | 0.69 |
| HbA1c, % | 5.94 (0.79) | 5.94 (0.81) | | 5.95 (0.78) | | | 5.96 (0.80) | 5.83 (0.51) | | | 0.78 |
| Total Cholesterol, mg/dL | 152.52 (47.93) | 150.58 (48.15) | | 157.27 (47.33) | | | 152.80 (42.33) | 181.50 (65.17) | | | 0.064 |
| LDL, mg/dL | 82.54 (38.79) | 82.22 (40.18) | | 83.21 (35.95) | | | 80.95 (34.44) | 100.84 (44.24) | | | 0.31 |
| HDL, mg/dL | 52.93 (16.18) | 50.21 (17.04) | | 55.77 (14.74) | | | 55.77 (14.50) | 55.80 (19.22) | | | **0.005** |
| Lipoprotein(a), mg/dL | 67.02 (96.32) | 74.36 (107.29) | | 49.66 (60.64) | | | 46.91 (59.17) | 78.80 (75.68) | | | 0.20 |
| Leukocytes, G/L | 7.17 (2.65) | 7.27 (3.15) | | 7.07 (2.04) | | | 7.04 (1.88) | 7.43 (3.48) | | | 0.59 |
| Hemoglobin, g/dL | 13.07 (1.97) | 12.51 (2.09) | | 13.63 (1.67) | | | 13.75 (1.61) | 12.21 (1.78) | | | **<0.001** |
| CRP, mg/dl | 0.83 (1.83) | 1.14 (2.32) | | 0.52 (1.05) | | | 0.54 (1.09) | 0.30 (0.35) | | | **0.001** |
| **Concomitant medication** |  |  | |  | | |  |  | | |  |
| Beta-blocker, % | 58.54% | | 71.43% | | 53.20% | 52.55% | | | 71.43% | **0.010** | |
| ACE-inhibitors, % | 21.20% | | 28.05% | | 18.41% | 18.46% | | | 16.67% | 0.20 | |
| ARBs, % | 29.12% | | 41.46% | | 24.14% | 23.98% | | | 28.57% | **0.014** | |
| Calcium channel blockers, % | 17.25% | | 27.16% | | 13.30% | 12.76% | | | 28.57% | **0.011** | |
| Statin, % | 46.58% | | 50.00% | | 45.19% | 45.50% | | | 37.50% | 0.69 | |
| T-ASS, % | 31.12% | | 45.24% | | 25.25% | 24.49% | | | 50.00% | **0.002** | |
| Coumarin, % | 23.21% | | 21.95% | | 26.67% | 25.00% | | | 50.00% | 0.63 | |
| NOAC, % | 34.45% | | 28.05% | | 48.65% | 47.06% | | | 66.67% | 0.072 | |
| Diuretics, % | 51.05% | | 57.14% | | 48.51% | 47.45% | | | 83.33% | 0.092 | |

*Data are presented as mean ± SD or n (%). P-values reflect pairwise comparisons between CA patients and controls in the propensity score-matched cohort, using independent t-tests for continuous variables and chi-square tests for categorical variables. Excellent balance (SMD < 0.1) was achieved for 4 of 5 variables. Standardized mean differences (SMD) <0.1 indicates excellent balance (achieved for 4/5 variables). Log NT-proBNP showed a slightly higher SMD of 0.117, representing an 82.6% reduction in bias compared with the unmatched cohort.*

AP, alkaline phosphatase; ALT, alanine aminotransferase; AST, aspartate aminotransferase; BSA, body surface area; BMI, body mass index; CAD, coronary artery disease; CMR, cardiac magnetic resonance; CK-MB, creatine kinase myocardial band; CRP, C-reactive protein; eGFR, estimated glomerular filtration rate; gGT, gamma-glutamyl transferase; HbA1c, glycated hemoglobin; HF, heart failure; INR, international normalized ratio; LA, left atrium; LDL, low-density lipoprotein cholesterol; LVEF, left ventricular ejection fraction; LVEDV, left ventricular end-diastolic volume; LVESV, left ventricular end-systolic volume; LVCO, left ventricular cardiac output; LVSV, left ventricular stroke volume; NT-proBNP, N-terminal prohormone of brain natriuretic peptide; RA, right atrium; RVEF, right ventricular ejection fraction; RVEDV, right ventricular end-diastolic volume; RVESV, right ventricular end-systolic volume; RVCO, right ventricular cardiac output; sPAP, estimated systolic pulmonary artery pressure; TnT, troponin T; VHD, valvular heart disease; T-ASS, thrombo-embolic prophylaxis (acetylsalicylic acid); NOAC, non-vitamin K oral anticoagulants; ACE, angiotensin-converting enzyme; ARB, angiotensin receptor blocker. Bold values indicate p<0.05.

**Supplementary Table 3.** Baseline CMR characteristics of controls and CA subtypes after propensity score matching

|  | **Total** | **Controls** | **CA** | **ATTR-CA** | **AL-CA** | **p-value** |
| --- | --- | --- | --- | --- | --- | --- |
|  | N=470 | N=235 | N=235 | N=217 | N=18 |  |
| CMR parameters |  |  |  |  |  |  |
| LV mass, g | 174.46 (55.18) | 163.25 (52.40) | 184.13 (55.81) | 184.18 (55.48) | 183.50 (62.69) | **<0.001** |
| LVEDV, mL | 172.53 (55.34) | 180.54 (64.21) | 164.94 (44.17) | 166.91 (44.72) | 139.86 (26.25) | **0.002** |
| LVEDV/BSA, mL/m^2^ | 90.33 (29.26) | 93.38 (32.05) | 84.69 (22.30) | 85.90 (23.16) | 76.39 (12.74) | **0.017** |
| LVEF, % | 51.56 (13.76) | 52.80 (15.63) | 50.40 (11.66) | 50.10 (11.67) | 54.14 (11.23) | 0.091 |
| LVSV, mL | 85.34 (25.99) | 89.17 (26.74) | 81.69 (24.77) | 82.25 (25.14) | 74.12 (18.07) | **0.004** |
| LVSV/BSA, mL/m^2^ | 44.25 (13.10) | 46.03 (13.36) | 40.94 (11.96) | 41.00 (12.33) | 40.56 (9.32) | **0.003** |
| IVS, mm | 15.81 (4.64) | 12.91 (3.04) | 18.70 (4.12) | 18.83 (4.05) | 16.94 (4.77) | **<0.001** |
| LVCO, mL | 5.82 (5.58) | 5.57 (1.68) | 6.06 (7.62) | 6.17 (7.88) | 4.59 (1.19) | 0.36 |
| LVCO/BSA, mL/m^2^ | 2.96 (3.12) | 2.88 (0.87) | 3.09 (5.14) | 3.19 (5.50) | 2.47 (0.66) | 0.59 |
| RVEDV, mL | 173.06 (54.45) | 172.48 (58.65) | 173.61 (50.28) | 174.79 (50.93) | 158.68 (39.18) | 0.49 |
| RVEDV/BSA, mL/m^2^ | 90.09 (27.64) | 88.68 (28.21) | 92.70 (26.47) | 93.48 (27.40) | 87.31 (18.77) | 0.32 |
| RVEF, % | 48.31 (11.74) | 49.93 (12.01) | 46.77 (11.28) | 46.79 (11.19) | 46.49 (12.80) | **0.016** |
| RVSV, mL | 81.47 (26.30) | 83.47 (27.67) | 79.58 (24.85) | 80.23 (25.24) | 70.75 (16.97) | 0.11 |
| RVSV/BSA, mL/m^2^ | 41.68 (13.17) | 42.93 (13.44) | 39.37 (12.37) | 39.48 (12.87) | 38.65 (8.45) | 0.059 |
| RVCO, mL | 5.82 (5.58) | 5.57 (1.68) | 6.06 (7.62) | 5.56 (4.41) | 3.98 (1.34) | 0.13 |
| RVCO/BSA, mL/m^2^ | 2.96 (3.12) | 2.88 (0.87) | 3.09 (5.14) | 2.81 (2.84) | 2.13 (0.75) | 0.35 |
| LA volume, mL | 39.48 (6.20) | 39.86 (6.68) | 38.79 (5.21) | 38.73 (5.26) | 39.20 (5.04) | 0.30 |
| LA volume/BSA, mL/m^2^ | 20.66 (3.70) | 20.69 (4.01) | 20.62 (3.05) | 20.46 (3.10) | 21.73 (2.54) | 0.46 |
| RA volume, mL | 39.65 (15.47) | 37.73 (7.70) | 42.91 (23.15) | 39.98 (7.04) | 60.94 (59.90) | **<0.001** |
| RA volume/BSA, mL/m^2^ | 20.28 (8.98) | 18.81 (3.84) | 22.68 (13.47) | 20.85 (3.60) | 32.88 (33.68) | **<0.001** |
| Myocardial native T1, ms | 1062.78 (66.61) | 1026.97 (48.36) | 1098.59 (63.09) | 1096.94 (63.02) | 1118.39 (62.29) | **<0.001** |
| Blood native T1, ms | 1632.35 (128.53) | 1655.72 (139.67) | 1608.97 (111.82) | 1601.75 (107.77) | 1696.11 (125.78) | **<0.001** |
| Myocardial ECV, % | 38.05 (13.71) | 28.65 (6.05) | 47.13 (12.90) | 47.26 (12.62) | 45.53 (16.44) | **<0.001** |
| Muscle native T1, ms | 900.87 (68.16) | 887.86 (63.39) | 913.87 (70.37) | 912.34 (69.29) | 932.33 (82.32) | **<0.001** |
| Muscle ECV, % | 15.26 (4.81) | 14.17 (3.99) | 15.83 (5.10) | 15.45 (4.72) | 20.77 (7.22) | **<0.001** |

*Data are presented as mean ± SD or n (%). P-values reflect pairwise comparisons between CA patients and controls in the propensity score-matched cohort using independent t-tests for continuous variables and chi-square tests for categorical variables. Standardized mean differences (SMD) <0.1 indicates excellent balance (achieved for 4/5 variables). Log NT-proBNP showed a slightly higher SMD of 0.117, representing an 82.6% reduction in bias compared with the unmatched cohort.*

BSA indicates body surface area; BMI, body mass index; LV, left ventricle; LVEF, left ventricular ejection fraction; LVEDV, left ventricular end-diastolic volume; LVSV, left ventricular stroke volume; LVCO, left ventricular cardiac output; IVS, interventricular septum; RV, right ventricle; RVEF, right ventricular ejection fraction; RVEDV, right ventricular end-diastolic volume; RVSV, right ventricular stroke volume; RVCO, right ventricular cardiac output; LA, left atrium; RA, right atrium; ECV, extracellular volume; CA, cardiac amyloidosis; CMR, cardiac magnetic resonance; NT-proBNP, N-terminal prohormone of brain natriuretic peptide; HbA1C, glycated hemoglobin; AP, alkaline phosphatase; AST, aspartate aminotransferase; ALT, alanine aminotransferase; gGT, gamma-glutamyl transferase. Bold values indicate p<0.05.

**Supplementary Table 4.** Diagnostic performance of native thoracic skeletal muscle T1 for detection of cardiac amyloidosis across clinically relevant subgroups

| **Analysis** | **CA (n)** | **Controls (n)** | **AUC (95% CI)** | **p-value** |
| --- | --- | --- | --- | --- |
| **Overall** | 267 | 1,709 | 0.70 (0.66-0.73) | **<0.001** |
| **Matched cohorts** |  |  |  |  |
| Propensity score-matched | 234 | 114 | 0.68 (0.62-0.74) | **<0.001** |
| Age and sex-matched | 234 | 234 | 0.62 (0.57-0.68) | **<0.001** |
| **Clinically relevant comparator groups** |  |  |  |  |
| CA vs LVH (IVS >14 mm) | 237 | 187 | 0.67 (0.60-0.72) | **<0.001** |
| CA vs HF referrals ^*^ | 237 | 589 | 0.59 (0.54-0.64) | **<0.001** |
| CA vs elderly (age ≥70 years) | 198 | 610 | 0.61 (0.57-0.65) | **<0.001** |
| **Demographic strata** |  |  |  |  |
| Males | 213 | 928 | 0.72 (0.68-0.76) | **<0.001** |
| Females | 54 | 781 | 0.71 (0.64-0.79) | **<0.001** |
| Age <75 years | 109 | 1,269 | 0.70 (0.65-0.75) | **<0.001** |
| Age ≥75 years | 158 | 440 | 0.64 (0.59-0.69) | **<0.001** |
| **Covariate-adjusted** |  |  |  |  |
| Adjusted for age, sex, BSA | 267 | 1,709 | 0.83 (0.79-0.87) | **<0.001** |

^*^ Heart failure defined as left ventricular ejection fraction <50% or NT-proBNP >400 pg/mL.

Abbreviations: AUC indicates area under the curve; BSA, body surface area; CA, cardiac amyloidosis; CI, confidence interval; HF, heart failure; LVH, left ventricular hypertrophy, n, number; IVS, interventricular septum.
